# Epidemiology and patterns of tracheostomy practice in patients with acute respiratory distress syndrome in ICUs across 50 countries

**DOI:** 10.1186/s13054-018-2126-6

**Published:** 2018-08-17

**Authors:** Toshikazu Abe, Fabiana Madotto, Tài Pham, Isao Nagata, Masatoshi Uchida, Nanako Tamiya, Kiyoyasu Kurahashi, Giacomo Bellani, John G. Laffey, for the LUNG-SAFE Investigators and the ESICM Trials Group, Guy M. Francois, Francesca Rabboni, Fabiana Madotto, Sara Conti, John G. Laffey, Giacomo Bellani, Tai Pham, Eddy Fan, Antonio Pesenti, Laurent Brochard, Andres Esteban, Luciano Gattinoni, Frank van Haren, Anders Larsson, Daniel F. McAuley, Marco Ranieri, Gordon Rubenfeld, B. Taylor Thompson, Hermann Wrigge, Arthur S. Slutsky, Fernando Rios, T. Sottiaux, P. Depuydt, Fredy S. Lora, Luciano Cesar Azevedo, Guillermo Bugedo, Haibo Qiu, Marcos Gonzalez, Juan Silesky, Vladimir Cerny, Jonas Nielsen, Manuel Jibaja, Tài Pham, Dimitrios Matamis, Jorge Luis Ranero, Pravin Amin, S. M. Hashemian, Kevin Clarkson, Kiyoyasu Kurahashi, Asisclo Villagomez, Amine Ali Zeggwagh, Leo M. Heunks, Jon Henrik Laake, Jose Emmanuel Palo, Antero do Vale Fernandes, Dorel Sandesc, Yaasen Arabi, Vesna Bumbasierevic, Nicolas Nin, Jose A. Lorente, Lise Piquilloud, Fekri Abroug, Lia McNamee, Javier Hurtado, Ed Bajwa, Gabriel Démpair, Hektor Sula, Lordian Nunci, Alma Cani, Alan Zazu, Christian Dellera, Carolina S. Insaurralde, Risso V. Alejandro, Julio Daldin, Mauricio Vinzio, Ruben O. Fernandez, Luis P. Cardonnet, Lisandro R. Bettini, Mariano Carboni Bisso, Emilio M. Osman, Mariano G. Setten, Pablo Lovazzano, Javier Alvarez, Veronica Villar, Cesar Milstein, Norberto C. Pozo, Nicolas Grubissich, Gustavo A. Plotnikow, Daniela N. Vasquez, Santiago Ilutovich, Norberto Tiribelli, Ariel Chena, Carlos A. Pellegrini, María G. Saenz, Elisa Estenssoro, Matias Brizuela, Hernan Gianinetto, Pablo E. Gomez, Valeria I. Cerrato, Marco G. Bezzi, Silvina A. Borello, Flavia A. Loiacono, Adriana M. Fernandez, Serena Knowles, Claire Reynolds, Deborah M. Inskip, Jennene J. Miller, Jing Kong, Christina Whitehead, Shailesh Bihari, Aylin Seven, Amanda Krstevski, Helen J. Rodgers, Rebecca T. Millar, Toni E. Mckenna, Irene M. Bailey, Gabrielle C. Hanlon, Anders Aneman, Joan M. Lynch, Raman Azad, John Neal, Paul W. Woods, Brigit L. Roberts, Mark R. Kol, Helen S. Wong, Katharina C. Riss, Xavier Wittebole, Caroline Berghe, Pierre A. Bulpa, Alain M. Dive, Rik Verstraete, Herve Lebbinck, Pieter Depuydt, Joris Vermassen, Philippe Meersseman, Helga Ceunen, Jonas I. Rosa, Daniel O. Beraldo, Claudio Piras, Adenilton M. Rampinelli, Antonio P. Nassar, Sergio Mataloun, Marcelo Moock, Marlus M. Thompson, Claudio H. Gonçalves, Ana Carolina P. Antônio, Aline Ascoli, Rodrigo S. Biondi, Danielle C. Fontenele, Danielle Nobrega, Vanessa M. Sales, Suresh Shindhe, Dayangku Hajah Maizatul Aiman binti Pengiran Haji Ismail, John Laffey, Francois Beloncle, Kyle G. Davies, Rob Cirone, Venika Manoharan, Mehvish Ismail, Ewan C. Goligher, Mandeep Jassal, Erin Nishikawa, Areej Javeed, Gerard Curley, Nuttapol Rittayamai, Matteo Parotto, Niall D. Ferguson, Sangeeta Mehta, Jenny Knoll, Antoine Pronovost, Sergio Canestrini Chile, Alejandro R. Bruhn, Patricio H. Garcia, Felipe A. Aliaga, Pamela A. Farías, Jacob S. Yumha, Claudia A. Ortiz, Javier E. Salas, Alejandro A. Saez, Luis D. Vega, Eduardo F. Labarca, Felipe T. Martinez, Nicolás G. Carreño, Pilar Lora, Haitao Liu, Ling Liu, Rui Tang, Xiaoming Luo, Youzhong An, Huiying Zhao, Yan Gao, Zhe Zhai, Zheng L. Ye, Wei Wang, Wenwen Li, Qingdong Li, Ruiqiang Zheng, Wenkui Yu, Juanhong Shen, Xinyu Li, Tao Yu, Weihua Lu, Ya Q. Wu, Xiao B. Huang, Zhenyang He, Yuanhua Lu, Hui Han, Fan Zhang, Renhua Sun, Hua X. Wang, Shu H. Qin, Bao H. Zhu, Jun Zhao, Jian Liu, Bin Li, Jing L. Liu, Fa C. Zhou, Qiong J. Li, Xing Y. Zhang, Zhou Li-Xin, Qiang Xin-Hua, Liangyan Jiang, Yuan N. Gao, Xian Y. Zhao, Yuan Y. Li, Xiao L. Li, Chunting Wang, Qingchun Yao, Rongguo Yu, Kai Chen, Huanzhang Shao, Bingyu Qin, Qing Q. Huang, Wei H. Zhu, Ai Y. Hang, Ma X. Hua, Yimin Li, Yonghao Xu, Yu D. Di, Long L. Ling, Tie H. Qin, Shou H. Wang, Junping Qin, Yi Han, Monica P. Vargas, Juan I. Silesky Jimenez, Manuel A. González Rojas, Jaime E. Solis-Quesada, Christian M. Ramirez-Alfaro, Jan Máca, Peter Sklienka, Jakob Gjedsted, Boris G. Villamagua, Miguel Llano, Philippe Burtin, Gautier Buzancais, Pascal Beuret, Nicolas Pelletier, Satar Mortaza, Alain Mercat, Jonathan Chelly, Sébastien Jochmans, Nicolas Terzi, Cédric Daubin, Guillaume Carteaux, Nicolas de Prost, Jean-Daniel Chiche, Fabrice Daviaud, Muriel Fartoukh, Guillaume Barberet, Jerome Biehler, Jean Dellamonica, Denis Doyen, Jean-Michel Arnal, Anais Briquet, Sami Hraiech, Laurent Papazian, Damien Roux, Jonathan Messika, Evangelos Kalaitzis, Réanimation Médicale, Laurence Dangers, Alain Combes, Siu-Ming Au, Gaetan Béduneau, Dorothée Carpentier, Elie H. Zogheib, Herve Dupont, Sylvie Ricome, Francesco L. Santoli, Sebastien L. Besset, Philippe Michel, Bruno Gelée, Pierre-Eric Danin, Bernard Goubaux, Philippe J. Crova, Nga T. Phan, Frantz Berkelmans, Julio C. Badie, Romain Tapponnier, Josette Gally, Samy Khebbeb, Jean-Etienne Herbrecht, Francis Schneider, Pierre-Louis M. Declercq, Jean-Philippe Rigaud, Jacques Duranteau, Anatole Harrois, Russell Chabanne, Julien Marin, Charlene Bigot, Sandrine Thibault, Mohammed Ghazi, Messabi Boukhazna, Salem Ould Zein, Jack R. Richecoeur, Daniele M. Combaux, Fabien Grelon, Charlene Le Moal, Elise P. Sauvadet, Adrien Robine, Virginie Lemiale, Danielle Reuter, Martin Dres, Alexandre Demoule, Dany Goldgran-Toledano, Loredana Baboi, Claude Guérin, Ralph Lohner, Jens Kraßler, Susanne Schäfer, Kai D. Zacharowski, Patrick Meybohm, Andreas W. Reske, Philipp Simon, Hans-Bernd F. Hopf, Michael Schuetz, Thomas Baltus, Metaxia N. Papanikolaou, Theonymfi G. Papavasilopoulou, Giannis A. Zacharas, Vasilis Ourailogloy, Eleni K. Mouloudi, Eleni V. Massa, Eva O. Nagy, Electra E. Stamou, Ellada V. Kiourtzieva, Marina A. Oikonomou, Luis E. Avila, Cesar A. Cortez, Johanna E. Citalán, Sameer A. Jog, Safal D. Sable, Bhagyesh Shah, Mohan Gurjar, Arvind K. Baronia, Mohammedfaruk Memon, Radhakrishnan Muthuchellappan, Venkatapura J. Ramesh, Anitha Shenoy, Ramesh Unnikrishnan, Subhal B. Dixit, Rachana V. Rhayakar, Nagarajan Ramakrishnan, Vallish K. Bhardwaj, Heera L. Mahto, Sudha V. Sagar, Vijayanand Palaniswamy, Deeban Ganesan, Seyed Mohammadreza Hashemian, Hamidreza Jamaati, Farshad Heidari, Edel A. Meaney, Alistair Nichol, Karl M. Knapman, Donall O’Croinin, Eimhin S. Dunne, Dorothy M. Breen, Kevin P. Clarkson, Rola F. Jaafar, Rory Dwyer, Fahd Amir, Olaitan O. Ajetunmobi, Aogan C. O’Muircheartaigh, Colin S. Black, Nuala Treanor, Daniel V. Collins, Wahid Altaf, Gianluca Zani, Maurizio Fusari, Savino Spadaro, Carlo A. Volta, Romano Graziani, Barbara Brunettini, Salvatore Palmese, Paolo Formenti, Michele Umbrello, Andrea Lombardo, Elisabetta Pecci, Marco Botteri, Monica Savioli, Alessandro Protti, Alessia Mattei, Lorenzo Schiavoni, Andrea Tinnirello, Manuel Todeschini, Antonino Giarratano, Andrea Cortegiani, Sara Sher, Anna Rossi, Massimo M. Antonelli, Luca M. Montini, Paolo Casalena, Sergio Scafetti, Giovanna Panarello, Giovanna Occhipinti, Nicolò Patroniti, Matteo Pozzi, Roberto R. Biscione, Michela M. Poli, Ferdinando Raimondi, Daniela Albiero, Giulia Crapelli, Eduardo Beck, Vincenzo Pota, Vincenzo Schiavone, Alexandre Molin, Fabio Tarantino, Giacomo Monti, Elena Frati, Lucia Mirabella, Gilda Cinnella, Tommaso Fossali, Riccardo Colombo, Pierpaolo Terragni Ilaria Pattarino, Francesco Mojoli, Antonio Braschi, Erika E. Borotto, Andrea N. Cracchiolo, Daniela M. Palma, Francesco Raponi, Giuseppe Foti, Ettore R. Vascotto, Andrea Coppadoro, Luca Brazzi, Leda Floris, Giorgio A. Iotti, Aaron Venti, Osamu Yamaguchi, Shunsuke Takagi, Hiroki N. Maeyama, Eizo Watanabe, Yoshihiro Yamaji, Kazuyoshi Shimizu, Kyoko Shiozaki, Satoru Futami, Sekine Ryosuke, Koji Saito, Yoshinobu Kameyama, Keiko Ueno, Masayo Izawa, Nao Okuda, Hiroyuki Suzuki, Tomofumi Harasawa, Michitaka Nasu, Tadaaki Takada, Fumihito Ito, Shin Nunomiya, Kansuke Koyama, Toshikazu Abe, Kohkichi Andoh, Kohei Kusumoto, Akira Hirata, Akihiro Takaba, Hiroyasu Kimura, Shuhei Matsumoto, Ushio Higashijima, Hiroyuki Honda, Nobumasa Aoki, Hiroshi Imai, Yasuaki Ogino, Ichiko Mizuguchi, Kazuya Ichikado, Kenichi Nitta, Katsunori Mochizuki, Tomoaki Hashida, Hiroyuki Tanaka, Tomoyuki Nakamura, Daisuke Niimi, Takeshi Ueda, Yozo Kashiwa, Akinori Uchiyama, Olegs Sabelnikovs, Peteris Oss Lebanon, Youssef Haddad, Kong Y. Liew, Silvio A. Ñamendys-Silva, Yves D. Jarquin-Badiola, Luis A. Sanchez-Hurtado, Saira S. Gomez-Flores, Maria C. Marin, Asisclo J. Villagomez, Hospital General, Jordana S. Lemus, Jonathan M. Fierro, Mavy Ramirez Cervantes, Francisco Javier Flores Mejia, Dulce Dector, Dulce M. Dector, Daniel R. Gonzalez, Claudia R. Estrella, Jorge R. Sanchez-Medina, Alvaro Ramirez-Gutierrez, Fernando G. George, Janet S. Aguirre, Juan A. Buensuseso, Manuel Poblano, Tarek Dendane, Hicham Balkhi, Mina Elkhayari, Nacer Samkaoui, Hanane Ezzouine, Abdellatif Benslama, Mourad Amor, Wajdi Maazouzi, Nedim Cimic, Oliver Beck, Monique M. Bruns, Jeroen A. Schouten, Myra Rinia, Monique Raaijmakers, Hellen M. Van Wezel, Serge J. Heines, Ulrich Strauch, Marc P. Buise, Fabienne D. Simonis, Marcus J. Schultz, Jennifer C. Goodson, Troy S. Browne, Leanlove Navarra, Anna Hunt, Robyn A. Hutchison, Mathew B. Bailey, Lynette Newby, Colin Mcarthur, Michael Kalkoff, Alex Mcleod, Jonathan Casement, Danielle J. Hacking, Finn H. Andersen, Merete S. Dolva, Jon H. Laake, Andreas Barratt-Due, Kim Andre L. Noremark, Eldar Søreide, Brit Å. Sjøbø, Anne B. Guttormsen, Hector H. Leon Yoshido, Ronald Zumaran Aguilar, Fredy A. Montes Oscanoa, Alain U. Alisasis, Joanne B. Robles, Rossini Abbie B. Pasanting-Lim, Beatriz C. Tan Poland, Pawel Andruszkiewicz, Karina Jakubowska, Cristina M. Coxo, António M. Alvarez, Bruno S. Oliveira, Gustavo M. Montanha, Nelson C. Barros, Carlos S. Pereira, António M. Messias, Jorge M. Monteiro, Ana M. Araujo, Nuno T. Catorze, Susan M. Marum, Maria J. Bouw, Rui M. Gomes, Vania A. Brito, Silvia Castro, Joana M. Estilita, Filipa M. Barros, Isabel M. Serra, Aurelia Romania, Dana R. Tomescu, Alexandra Marcu, Ovidiu H. Bedreag, Marius Papurica, Dan E. Corneci, Silvius Ioan Negoita, Evgeny Grigoriev, Alexey I. Gritsan, Andrey A. Gazenkampf, Ghaleb Almekhlafi, Mohamad M. Albarrak, Ghanem M. Mustafa, Khalid A. Maghrabi, Nawal Salahuddin, Tharwat M. Aisa, Ahmed S. Al Jabbary, Edgardo Tabhan, Yaseen M. Arabim, Yaseen M. Arabi, Olivia A. Trinidad, Hasan M. Al Dorzi, Edgardo E. Tabhan, Stefan Bolon, Oliver Smith, Jordi Mancebo, Hernan Aguirre-Bermeo, Juan C. Lopez-Delgado, Francisco Esteve, Gemma Rialp, Catalina Forteza, Candelaria de Haro, Antonio Artigas, Guillermo M. Albaiceta, Sara de Cima-Iglesias, Leticia Seoane-Quiroga, Alexandra Ceniceros-Barros, Antonio L. Ruiz-Aguilar, Luis M. Claraco-Vega, Juan Alfonso Soler, Maria del Carmen Lorente, Cecilia Hermosa, Federico Gordo, Miryam Prieto-González, Juan B. López-Messa, Manuel P. Perez, Cesar P. Perez, Raquel Montoiro Allue, Ferran Roche-Campo, Marco Ibañez-Santacruz, Susana Temprano, Maria C. Pintado, Raul de Pablo, Pilar Ricart Aroa Gómez, Silvia Rodriguez Ruiz, Silvia Iglesias Moles, Maria Teresa Jurado, Alfons Arizmendi, Enrique A. Piacentini, Nieves Franco, Teresa Honrubia, Meisy Perez Cheng, Elena Perez Losada, Javier Blanco, Luis J. Yuste, Cecilia Carbayo-Gorriz, Francisca G. Cazorla-Barranquero, Javier G. Alonso, Rosa S. Alda, Ángela Algaba, Gonzalo Navarro, Enrique Cereijo, Esther Diaz-Rodriguez, Diego Pastor Marcos, Laura Alvarez Montero, Luis Herrera Para, Roberto Jimenez Sanchez, Miguel Angel Blasco Navalpotro, Ricardo Diaz Abad, Raquel Montiel González, Dácil Parrilla Toribio, Alejandro G. Castro, Maria Jose D. Artiga, Oscar Penuelas, Tomas P. Roser, Moreno F. Olga, Elena Gallego Curto, Rocío Manzano Sánchez, Vallverdu P. Imma, Garcia M. Elisabet, Laura Claverias, Monica Magret, Ana M. Pellicer, Lucia L. Rodriguez, Jesús Sánchez-Ballesteros, Ángela González-Salamanca, Antonio G. Jimenez, Francisco P. Huerta, Juan Carlos J. Sotillo Diaz, Esther Bermejo Lopez, David D. Llinares Moya, Alec A. Tallet Alfonso, Palazon Sanchez Eugenio Luis, Palazon Sanchez Cesar, Sánchez I. Rafael, Corcoles G. Virgilio, Noelia N. Recio, Richard O. Adamsson, Christian C. Rylander, Bernhard Holzgraefe, Lars M. Broman, Joanna Wessbergh, Linnea Persson, Fredrik Schiöler, Hans Kedelv, Anna Oscarsson Tibblin, Henrik Appelberg, Lars Hedlund, Johan Helleberg, Karin E. Eriksson, Rita Glietsch, Niklas Larsson, Ingela Nygren, Silvia L. Nunes, Anna-Karin Morin, Thomas Kander, Anne Adolfsson, Hervé O. Zender, Corinne Leemann-Refondini, Souheil Elatrous, Slaheddine Bouchoucha, Imed Chouchene, Islem Ouanes, Asma Ben Souissi, Salma Kamoun, Oktay Demirkiran, Mustafa Aker, Emre Erbabacan, Ilkay Ceylan, Nermin Kelebek Girgin, Menekse Ozcelik, Necmettin Ünal, Basak Ceyda Meco, Onat O. Akyol, Suleyman S. Derman, Barry Kennedy, Ken Parhar, Latha Srinivasa, Danny McAuley, Phil Hopkins, Clare Mellis, Vivek Kakar, Dan Hadfield, Andre Vercueil, Kaushik Bhowmick, Sally K. Humphreys, Andrew Ferguson, Raymond Mckee, Ashok S. Raj, Danielle A. Fawkes, Philip Watt, Linda Twohey, Rajeev R. JhaMatthew Thomas, Alex Morton, Varsha Kadaba, Mark J. Smith, Anil P. Hormis, Santhana G. Kannan, Miriam Namih, Henrik Reschreiter, Julie Camsooksai, Alek Kumar, Szabolcs Rugonfalvi, Christopher Nutt, Orla Oneill, Colette Seasman, Ged Dempsey, Christopher J. Scott, Helen E. Ellis, Stuart Mckechnie, Paula J. Hutton, Nora N. Di Tomasso, Michela N. Vitale, Ruth Griffin, Michael N. Dean, Julius H. Cranshaw, Emma L. Willett, Nicholas Ioannou, Sarah Gillis, Peter Csabi, Rosaleen Macfadyen, Heidi Dawson, Pieter D. Preez, Alexandra J. Williams, Owen Boyd, Laura Ortiz-Ruiz de Gordoa, Jon Bramall, Sophie Symmonds, Simon K. Chau, Tim Wenham, Tamas Szakmany, Piroska Toth-Tarsoly, Katie H. Mccalman, Peter Alexander, Lorraine Stephenson, Thomas Collyer, Rhiannon Chapman, Raphael Cooper, Russell M. Allan, Malcolm Sim, David W. Wrathall, Donald A. Irvine, Kim S. Zantua, John C. Adams, Andrew J. Burtenshaw, Gareth P. Sellors, Ingeborg D. Welters, Karen E. Williams, Robert J. Hessell, Matthew G. Oldroyd, Ceri E. Battle, Suresh Pillai, Sinead C. Okane, Adrian Donnelly, Aniko D. Frigyik, Jon P. Careless, Martin M. May, Richard Stewart, T. John Trinder, Samantha J. Hagan, Matt P. Wise, Jade M. Cole, Caroline C. MacFie, Anna T. Dowling, Nicolás Nin, Edgardo Nuñez, Gustavo Pittini, Ruben Rodriguez, María C. Imperio, Cristina Santos, Ana G. França, Alejandro Ebeid, Alberto Deicas, Aditya Uppalapati, Ghassan Kamel, Valerie M. Banner-Goodspeed, Jeremy R. Beitler, Satyanarayana Reddy Mukkera, Shreedhar Kulkarni, Jarone Lee, Tomaz Mesar, John O. Shinn Iii, Dina Gomaa, Christopher Tainter, Dale J. Yeatts, Jessica Warren, Michael J. Lanspa, Russel R. Miller, Colin K. Grissom, Samuel M. Brown, Philippe R. Bauer, Ryan J. Gosselin, Barrett T. Kitch, Jason E. Cohen, Scott H. Beegle, Renaud M. Gueret, Aiman Tulaimat, Shazia Choudry, William Stigler, Hitesh Batra, Nidhi G. Huff, Keith D. Lamb, Trevor W. Oetting, Nicholas M. Mohr, Claine Judy, Shigeki Saito, Fayez M. Kheir, Fayez Kheir, Adam B. Schlichting, Angela Delsing, Daniel R. Crouch, Mary Elmasri, Dina Ismail, Kyle R. Dreyer, Thomas C. Blakeman, Dina Gomaa, Rebecca M. Baron, Carolina Quintana Grijalba, Peter C. Hou, Raghu Seethala, Imo Aisiku, Galen Henderson, Gyorgy Frendl, Sen-Kuang Hou, Robert L. Owens, Ashley Schomer, Vesna Bumbasirevic, Bojan Jovanovic, Maja Surbatovic, Milic Veljovic

**Affiliations:** 1 Department of General Medicine, Juntendo University, 2-1-1, Hongo, Bunkyo-ku, Tokyo, 113-0033 Japan; 2 Department of Health Services Research, University of Tsukuba, Tsukuba, Japan; 3 Research Center on Public Health, School of Medicine and Surgery, University of Milano-Bicocca, Monza, Italy; 4 Keenan Research Center for Biomedical Science, Li Ka Shing Knowledge Institute, St. Michael’s Hospital, Toronto, Canada; 5 Interdepartmental Division of Critical Care Medicine, University of Toronto, Toronto, Canada; 6 Department of Anesthesiology and Intensive Care Medicine, International University of Health and Welfare, School of Medicine, Narita, Japan; 7 Dipartimento di Medicina e Chirurgia, Università degli Studi Milano Bicocca, Milan, Italy; 8 Anesthesia, School of Medicine, National University of Ireland, Galway, Ireland

**Keywords:** Tracheostomy, Acute respiratory distress syndrome (ARDS), ICU, Ventilation, Propensity-matched analysis

## Abstract

**Background:**

To better understand the epidemiology and patterns of tracheostomy practice for patients with acute respiratory distress syndrome (ARDS), we investigated the current usage of tracheostomy in patients with ARDS recruited into the Large Observational Study to Understand the Global Impact of Severe Acute Respiratory Failure (LUNG-SAFE) study.

**Methods:**

This is a secondary analysis of LUNG-SAFE, an international, multicenter, prospective cohort study of patients receiving invasive or noninvasive ventilation in 50 countries spanning 5 continents. The study was carried out over 4 weeks consecutively in the winter of 2014, and 459 ICUs participated. We evaluated the clinical characteristics, management and outcomes of patients that received tracheostomy, in the cohort of patients that developed ARDS on day 1–2 of acute hypoxemic respiratory failure, and in a subsequent propensity-matched cohort.

**Results:**

Of the 2377 patients with ARDS that fulfilled the inclusion criteria, 309 (13.0%) underwent tracheostomy during their ICU stay. Patients from high-income European countries (*n* = 198/1263) more frequently underwent tracheostomy compared to patients from non-European high-income countries (*n* = 63/649) or patients from middle-income countries (*n* = 48/465). Only 86/309 (27.8%) underwent tracheostomy on or before day 7, while the median timing of tracheostomy was 14 (Q1–Q3, 7–21) days after onset of ARDS. In the subsample matched by propensity score, ICU and hospital stay were longer in patients with tracheostomy. While patients with tracheostomy had the highest survival probability, there was no difference in 60-day or 90-day mortality in either the patient subgroup that survived for at least 5 days in ICU, or in the propensity-matched subsample.

**Conclusions:**

Most patients that receive tracheostomy do so after the first week of critical illness. Tracheostomy may prolong patient survival but does not reduce 60-day or 90-day mortality.

**Trial registration:**

ClinicalTrials.gov, NCT02010073. Registered on 12 December 2013.

**Electronic supplementary material:**

The online version of this article (10.1186/s13054-018-2126-6) contains supplementary material, which is available to authorized users.

## Background

Tracheostomy is a widely used intervention in patients with acute respiratory failure, especially when clinicians predict a patient’s need for prolonged mechanical ventilation. This well-tolerated procedure reduces the requirement for sedation, results in better patient comfort, and facilitates earlier resumption of patient autonomy [1, 2]. On the other hand, tracheostomy carries risks of adverse events including procedure-related complications including death (albeit rare) and later cosmetic concerns [3]. The use of this procedure has increased over the last decade, in part because of the introduction of a practical bedside percutaneous tracheostomy technique.

Acute respiratory distress syndrome (ARDS) is a major cause of respiratory failure and presents significant clinical challenges. It accounts for about 10% of ICU admissions [4]. The Large observational study to understand the global impact of severe acute respiratory failure (LUNG-SAFE study) showed that this syndrome was both under-recognized and under-treated and associated with a high mortality rate [5]. In this study, tracheotomy was performed on 13% of the patients with ARDS [5]. However, few data are available on the current practice of tracheostomy in the ICU setting [6]. Studies examining tracheostomy practices have been confined to single countries [7], sometimes gathered in meta-analyses [8, 9]. There is a lack of detailed information on global patterns of the use of tracheostomy, patient characteristics, the management of patients with tracheostomy, and the outcomes of these patients [10]. The impact on clinical practice of the TracMan clinical trial [11], which showed no benefit for early compared to later tracheostomy, remains unclear. Given these issues, the aim of our study was to investigate by secondary analysis the current patterns of tracheostomy usage in patients with ARDS requiring invasive mechanical ventilation.

## Methods

### Design, setting, and participants

**Fig. 1:**
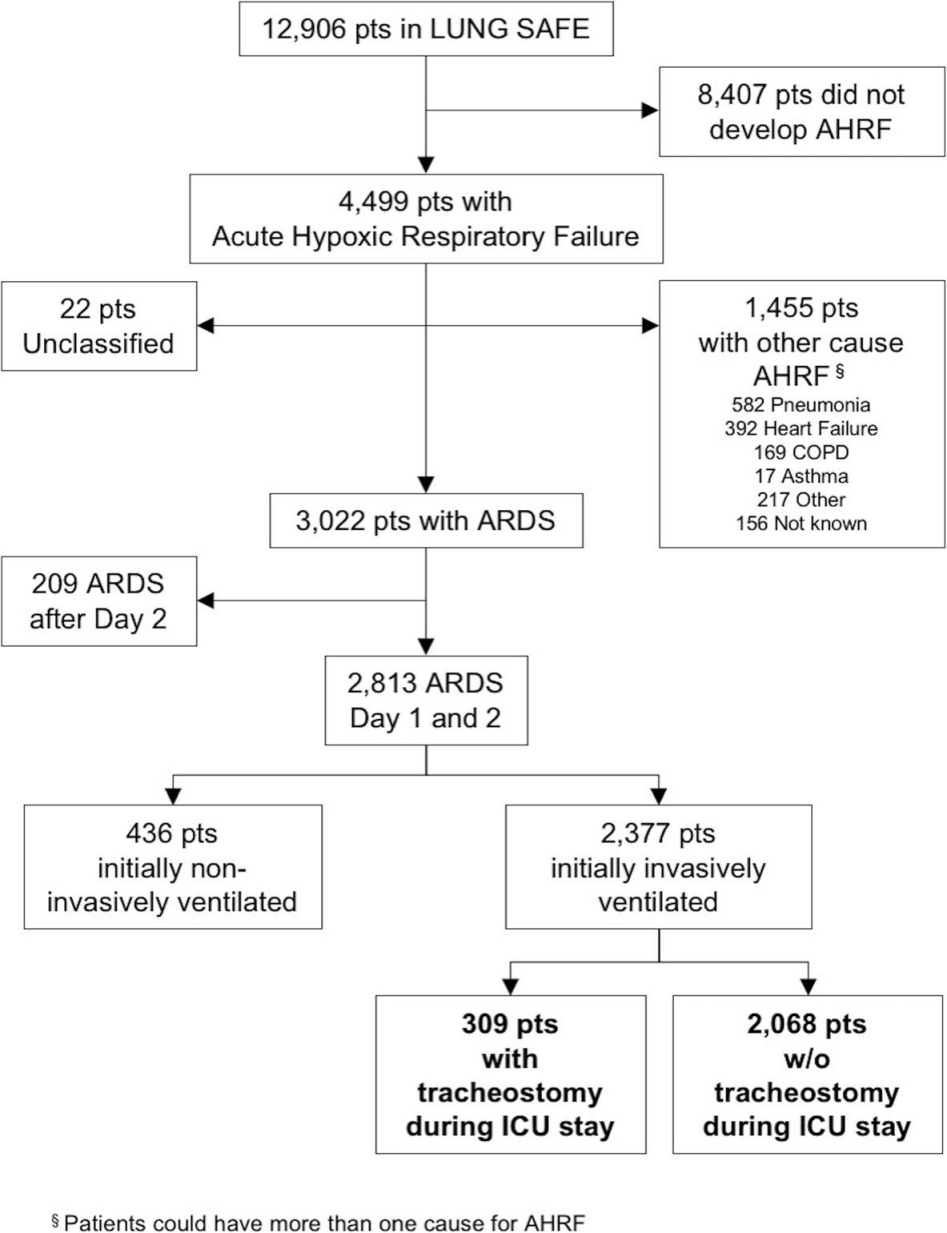
Flow chart of study participants. AHRF, acute hypoxic respiratory failure; ARDS, acute respiratory distress syndrome, Pts, patients; COPD, chronic obstructive pulmonary disease

This is a sub-study of the LUNG-SAFE study, an international, multicenter, prospective cohort study of patients receiving invasive or noninvasive ventilation. LUNG-SAFE used a convenience sample of 459 ICUs located in 50 countries, spanning 6 continents. The study was conducted over 4 weeks consecutively in each participating ICU in the winter of 2014 [5]. This study examined current use of tracheostomy in patients with ARDS requiring mechanical ventilation in ICUs. We included adult patients (≥ 16 years old) fulfilling ARDS criteria (according to the Berlin definition) who received invasive mechanical ventilation on day 1 or 2 from onset of acute hypoxemic respiratory failure (Fig. 1).

### Data collection and analysis

LUNG-SAFE is registered with ClinicalTrials.gov, number NCT02010073. Data were obtained from the LUNG-SAFE database, which was collected by the LUNG-SAFE investigators and the European Society of Intensive Care Medicine (ESICM) Trials Group [5]. Our study population was divided into two groups (tracheostomy and non-tracheostomy) according to whether tracheostomy was performed during the first 28 days in ICU after onset of acute hypoxemic respiratory failure. In each group, demographic factors, ARDS risk factors, patients’ comorbidities, illness severity, management factors such as ventilation setting measured on the day of ARDS onset, and outcomes that occurred during the ICU and hospital stay (days of mechanical ventilation, ventilator free days (VFDs), length of stay, and 28-day, 60-day, and 90-day mortality) were analyzed. In order to reduce the impact of the immortal time bias (i.e. bias due to fact that the patient had to be alive and still in the ICU to receive a tracheostomy) for tracheostomized patients, length of ICU and hospital stay, and 28-day, 60-day and 90-day mortality were calculated from the first day on which the investigator reported that the patient was tracheostomized. The impact of geo-economic location was also examined, with 3 areas defined: (1) European countries with high income, (2) non-European rest of world high-income countries (rWORLD), and (3) middle-income countries [10]. VFDs were defined as the number of days a patient was breathing without a ventilator during the 28-day study period, which began at the time of enrollment. Patients who died during the study period were assigned 0 for the number of VFDs. Since the amount of missing values was low, as previously reported [1], no assumptions were made for missing data.

Descriptive statistics included proportions for categorical variables and mean (standard deviation) or median (interquartile range (Q1–Q3)) for continuous variables. Comparisons between groups were assessed using the chi-square or Fisher exact test for discrete variables and Student *t* test or Wilcoxon rank-sum test for continuous variables, according to the data distribution (evaluated using the Shapiro-Wilk test). ICU and hospital mortality were evaluated at ICU or hospital discharge, or at day 90, whichever occurred first [4]. Survival analysis (Kaplan-Meier (K-M) approach) was performed to investigate the time to survival in patients with or without tracheostomy. We assumed that patients discharged alive from hospital before 90 days were alive on day 90. The difference in K-M curves between the groups was assessed using the log-rank test. We further evaluated the outcomes in the subgroup of patients who had ICU stays of at least 5 days duration (from acute hypoxemic respiratory failure onset), excluding those who died within 4 days, in order to reduce the potential for immortal time bias.

### Propensity-score matching

To assess the effect of tracheostomy on hospital mortality and other outcomes of interest and to reduce the potential for confounding by selection, we matched patients using the propensity-score matching approach. Logistic regression was used to estimate propensity scores able to predict the probability of undergoing tracheostomy. We included predictors that would affect the indication for tracheostomy (chosen a priori as possibly influencing the choice between tracheostomy or not): age, gender, and body mass index (BMI), region of enrollment, type of admission (medical, surgical planned or not, and trauma), comorbidities, ARDS risk factors (no risk factors, only direct risk factor, only indirect risk factor, both risk factors), use of extracorporeal memberane oxygenation (ECMO), arterial gas measures (pH, partial arterial pressure of oxygen (PaO_2_)/inspired fraction of oxygen (F_I_O_2_), and partial arterial pressure of carbon dioxide (PaCO_2_)) and non-respiratory sequential failure organ assessment (SOFA) score adjusted for missing values measured at date of ARDS onset. For tracheostomized patients, we used arterial blood gas and SOFA score measured on the last day before tracheostomy. Patients with similar propensity score in the two groups were matched (1:1 match without replacement), using a caliper of 0.2 standard deviation of the logit of the propensity score. We matched the data of the tracheostomized patient on one day before tracheostomy with those of a non-tracheostomized patient when they met the criteria for ARDS. We assessed the similarity of the matched groups through the standardized differences of each predictor. Statistical significance of the difference in means was evaluated by paired *t* test or Wilcoxon signed-rank test, while for the difference in proportions we applied McNemar’s test. The primary outcome was 90-day survival. The difference between the K-M survival curves in matched groups was assessed according to the test proposed by Klein and Moeschberger.

All *P* values were two-sided, with *P* values less than 0.05 considered as statistically significant. Statistical analyses were performed using SAS software, version 9.4 (SAS Institute, Cary, NC, USA) and R, version 3.3.3 (R Project for Statistical Computing, http://www.R-project.org).

## Results

Of the 2377 participants who were diagnosed with ARDS, according to the Berlin definition, on the 1st or 2nd day following development of acute hypoxemic respiratory failure and initially invasively ventilated, 309 (13.0%) underwent tracheostomy during their ICU stay (Fig. 1).

Patient demographics, including age, gender, and BMI did not differ between patients with or without tracheostomy (Table 1). There were significant variations with geo-economic region associated with the frequency of tracheostomy (*P* = 0.0002). High-income European areas had a greater frequency of tracheostomy than other areas. However, there was no significant difference in the frequency of undergoing tracheostomy between rWORLD countries and middle-income countries (*P* = 0.7353). Severity of ARDS at day 1 was also similar between the two groups (Table 1). Patients undergoing tracheostomy were more likely to have undergone elective surgery (Table 1), to have come from other hospital ICUs, have a lower frequency of chronic liver failure, and a higher frequency of pneumonia compared to patients that did not receive a tracheostomy (Additional file 1: Table S1). A large proportion of patients who received a tracheostomy received mechanical ventilation with spontaneous ventilator modes. Additional file 1 shows this in more detail. More patients who were on ECMO received tracheostomy compared to patients who were not on ECMO (Table 1).

**Table 1:** Baseline characteristics in patients with tracheostomy and patients with no tracheostomy. (*n* = 2377)

	Tracheostomy (*n* = 309)	No tracheostomy (*n* = 2068)	*P* value
	Number (%) or median (Q1-Q3)	Number (%) or median (Q1-Q3)	
Age (years)	63 (49–72)	63 (50–74)	0.1443
Sex (male)	200 (64.7)	1272 (61.5)	0.2775
BMI (kg/m^2^)	27.1 (23.1–30.8)	26.0 (22.9–30.4)	0.1410
Geo-economic area			0.0002
European countries with high income	198/1263 (15.7)	1065/1263 (84.3)	
Non-European countries (rest of world) with high income	63/649 (9.7)	586/649 (90.3)	
Countries with middle income	48/465 (10.3)	417/465 (89.7)	
Severity of ARDS at day 1			0.9271
Mild	95 (30.7)	619 (29.9)	
Moderate	144 (46.6)	962 (46.5)	
Severe	70 (22.7)	487 (23.6)	
Type of admission			0.0194
Medical	211 (68.3)	1554 (75.2)	
Surgical	20 (6.5)	123 (6.0)	
Postoperative (elective)	56 (18.1)	310 (15.0)	
Trauma	22 (7.1)	81 (4.0)	
Cause of AHRF			
Pneumonia	213 (68.9)	1295 (62.6)	0.0317
Cardiac failure	43 (12.9)	311 (15.0)	0.6051
Asthma	4 (1.3)	29 (1.4)	1.0000
ARDS (i.e. clinician recognized)	103 (33.3)	684 (33.1)	0.9284
COPD	29 (9.4)	205 (9.9)	0.7714
Unknown	15 (4.9)	117 (5.7)	0.5652
Others	54 (17.5)	418 (20.2)	0.2606
ARDS risk factor			0.1675
No risk factor	19 (6.1)	157 (7.6)	
Only indirect risk factors	53 (17.1)	429 (20.7)	
Only direct risk factors	194 (62.8)	1160 (56.1)	
Both risk factor types	43 (13.9)	322 (15.6)	
Illness severity at ARDS onset			
pH	7.37 (7.29–7.43)	7.34 (7.26–7.41)	<.0001
P/F ratio (mmHg)	156 (110–213)	154 (103–215)	0.6845
PaCO2 (mmHg)	42 (36–50)	43 (37–51)	0.2751
Non-respiratory SOFA score (adjusted for missing values) (ARDS onset)	6 (4–9)	7 (4–10)	0.0010
Non-respiratory SOFA score (adjusted for missing values) at day 2	6 (4–9)	6 (4–10)	0.1333
Difference in non-respiratory SOFA score (day2-day1) (adjusted for missing values)	0 (−2–2)	0 (−2–2)	0.9598
Mechanical ventilation settings at ARDS onset			
Respiratory rate (set) (breaths/min)	18 (15–22)	16 (14–20)	0.0016
Respiratory rate (total) (breaths/min)	20 (15–25)	20 (16–24)	0.2406
Tidal volume (ml)	488 (400–550)	457 (400–516)	0.0105
Tidal volume/IBW (ml/kg)	7.55 (6.34–8.96)	7.35 (6.39–8.48)	0.4519
PEEP (cmH2O)	8 (5–10)	8 (5–10)	0.0254
Plateau pressure (cmH2O) (*n* = 79, *n* = 663)	24 (20–27)	23 (18–28)	0.4957
Peak inspiratory pressure (cmH2O)	27 (21–32)	26 (22–32)	0.6444
Mean airway pressure (cmH2O)	14 (11–18)	14 (11–18)	0.4912
Use of adjuncts			
ECMO use	20 (6.5)	35 (1.7)	<.0001
High-dose corticosteroids	36 (11.7)	225 (10.9)	0.6862
Continuous sedation	234 (75.7)	1567 (75.8)	0.9861
Continuous neuromuscular blocking agents	34 (11.0)	286 (13.8)	0.1745
Renal replacement therapy	35 (11.3)	169 (8.2)	0.0648
Inhaled vasodilators	19 (6.2)	84 (4.1)	0.0928
Neutrophil elastase therapy	3 (1.0)	10 (0.5)	0.2334
Vasopressor used	163 (52.8)	1212 (58.6)	0.0518

*BMI* body mass index, *ICU* intensive care unit, *ER* emergency room, *COPD* chronic obstructive pulmonary disease, *NYHA* New York heart association, *AHRF* acute hypoxemic respiratory failure, *ARDS* acute respiratory distress syndrome, *TRALI* transfusion-related acute lung injury, *A/C* assist control, *PC* pressure control, *BIPAP* bilevel positive airway pressure, *APRV* airway pressure release ventilation, *SIMV* synchronized intermittent mandatory ventilation, *PRVC* pressure-regulated volume control, *PSV* pressure support ventilation, *HFO* high-frequency oscillation, *CPAP* continuous positive airway pressure, *IBW* ideal body weight, *PEEP* positive end-expiratory pressure, *ECMO* extracorporeal membrane oxygenation, *SOFA* sequential organ failure assessment, *PaCO2* partial arterial pressure of carbone dioxide

Missing data: BMI = 127; Ph = 25, PaCO2 = 25, respiratory rate (set) = 250, respiratory rate (total) = 10, tidal volume = 24, tidal volume/IBW = 122, peak inspiratory pressure = 89, mean airway pressure = 687, non-respiratory SOFA score (adjusted for missing values) = 22, non-respiratory SOFA score (adjusted for missing values) at day 2 = 379, difference in non-respiratory SOFA score (day2-day1) (adjusted for missing values) = 384

**Fig. 2:**
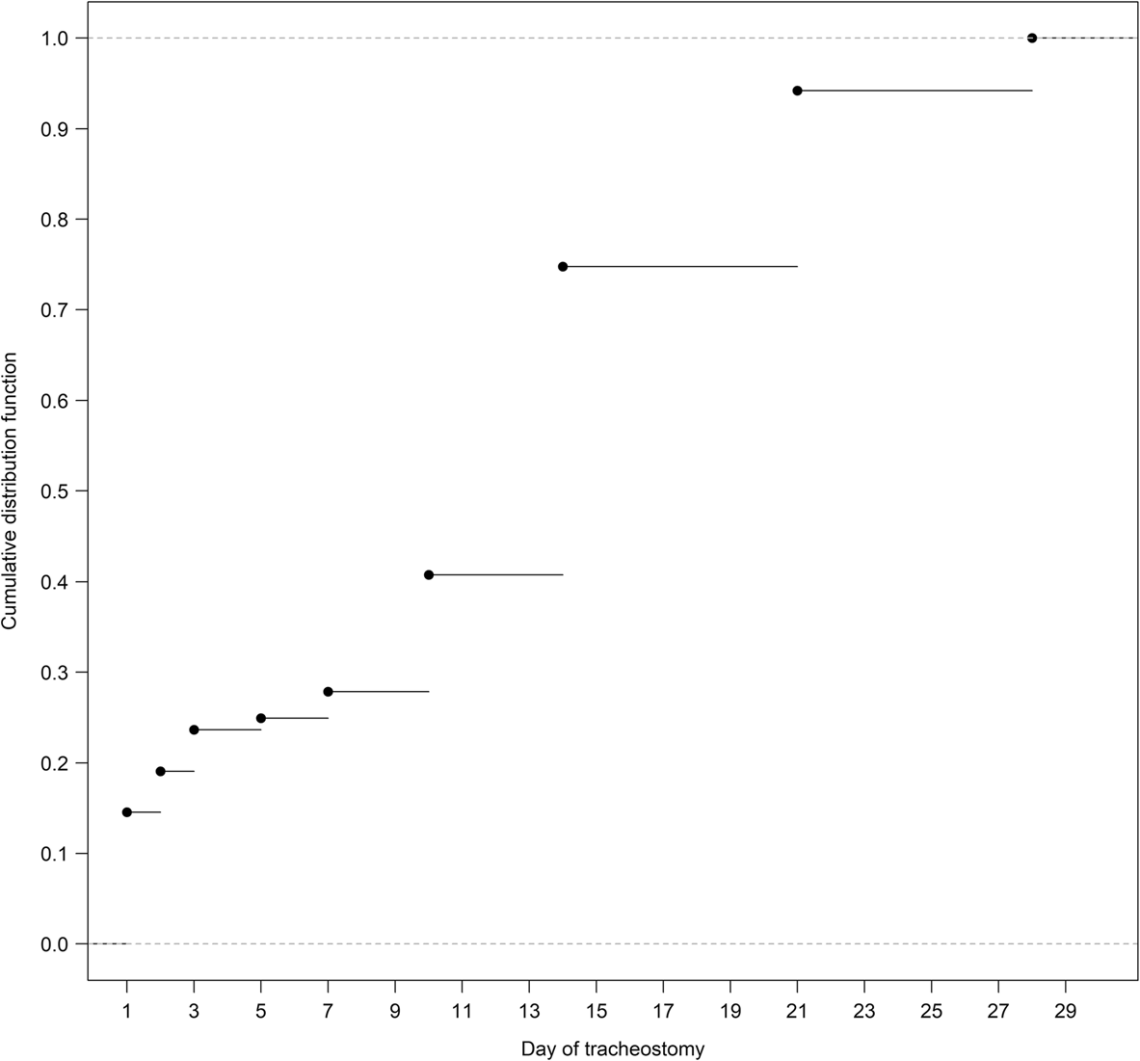
Distribution of time to tracheostomy (*n* = 309)

The median timing of tracheostomy was 14 (Q1–Q3, 7–21) days after onset of ARDS. Only 27.8% patients received tracheostomy within 7 days (Fig. 2), whereas 74.8% received it within 14 days. There was no difference in unadjusted outcome between patients receiving early versus late tracheostomy (Additional file 2: Table S2). Additional file 2 shows this in more detail.

The duration of mechanical ventilation in patients that received tracheostomy was significantly longer than that in those that did not (median (Q1–Q3) 21.5 (13–33) days vs 7 (4–13) days, *P* < 0.0001) (Table 2). Moreover, VFDs in the tracheostomy group was significantly shorter than in the non-tracheostomy group (median (Q1–Q3) 0 (0–13) days vs 15 (0–23) days, *P* < 0.0001). The length of ICU and hospital stay was also longer in patients that received tracheostomy (11 (5–23) days vs 8 (4–15) days, *P* < 0.0001 and 24 (9–44) days vs 14 (7–27) days, *P* < 0.0001, respectively).

**Table 2:** Outcomes in patients with tracheostomy and patients with no tracheostomy (*n* = 2377)

	Tracheostomy	No tracheostomy	*P* value
	Number (%) or median (Q1-Q3)	Number (%) or median (Q1-Q3)	
Days of mechanical ventilation			
All patients	21.5 (13–33)	7 (4–13)	<.0001
Patient alive at hospital discharge	21 (14–32)	7 (4–12)	<.0001
Ventilator-free days			
All patients	0 (0–13)	15 (0–23)	<.0001
Patient alive at hospital discharge (*n* = 181, *n* = 1114)	8 (0–15)	22 (17–25)	<.0001
Length of ICU stay (days)°			
All patients	11 (5–23)	8 (4–15)	<.0001
Patient alive at hospital discharge (*n* = 229, *n* = 1309)	12 (6–24)	9 (5–16)	0.0005
Length of hospital stay (days)°			
All patients	24 (9–44)	14 (7–27)	<.0001
Patient alive at hospital discharge (*n* = 200, *n* = 1165)	29.5 (15–50.5)	20 (12–35)	<.0001
Hospital mortality			
28-day* (*n* = 308, *n* = 2061)	72 (23.4)	786 (38.1)	<.0001
60-day* (*n* = 308, *n* = 2061)	91 (29.5)	847 (41.1)	0.0001
90-day* (*n* = 308, *n* = 2061)	95 (30.8)	861 (41.8)	0.0003
Limitation of life-sustaining therapies or measures decision (*n* = 308, *n* = 2061)	63 (20.4)	515 (24.9)	0.0844

*SD* standard deviation, *ICU* intensive care unit, *Q1–Q3* 25th–75th percentile

°For tracheostomized patients, length of stay was calculated from the “approximate” date of tracheostomy

*Mortality was evaluated according to the vital status at 28/60/90 days from acute respiratory distress syndreom onset or from the “nearest recorded” date of tracheostomy in non tracheostomized and tracheostomized patients, respectively. If the patient was discharged alive before 28/60/90 days, we considered the patient as alive

**Fig. 3:**
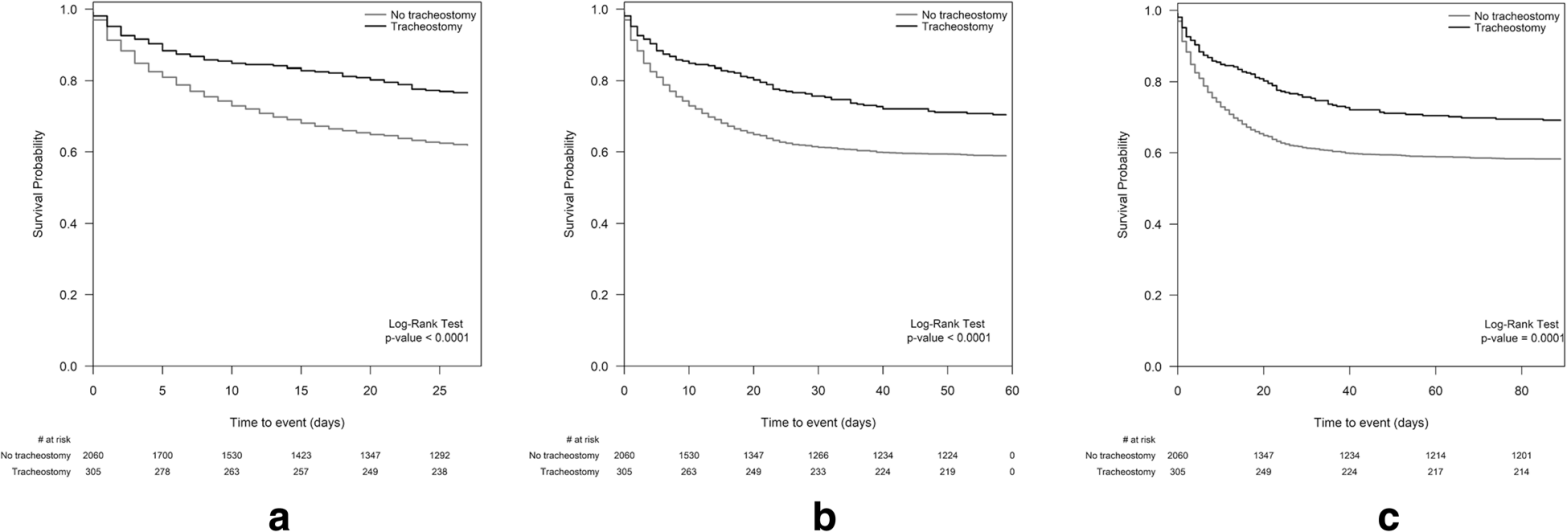
1 Survival probability during the first 28 days. 2 Survival probability during the first 60 days. 3 Survival probability during the first 90 days

The 28-day crude mortality in tracheostomized patients (23.4%) was lower than that in non-tracheostomized patients (38.1%). The 60-day and 90-day crude mortality in tracheostomized patients were both lower than that in non-tracheostomized patients (29.5% vs 41.1%, *P* = 0.0001 and 30.8% vs 41.8%, *P* = 0.0003, respectively). Survival analyses showed that, at any instance during the first 90 days after enrollment, tracheostomized patients were less likely to die than those in the non-tracheostomy group (30 days, *P* < 0.0001; 60 days *P* < 0.0001; 90 days, P = 0.0001; Table 2 and Fig. 3).

To reduce the impact of survivor bias, we analyzed the subgroup of patients who had been in the ICU for at least 5 days (*n* = 1670). Of these patients, 17.4% were tracheostomized (290 patients). Again, we found that the duration of mechanical ventilation in the tracheostomy group was significantly longer than that in the non-tracheostomy group (median (Q1–Q3) 22 (14–34) days versus 10 (7–16) days, *P* < 0.0001). Also, the length of hospital stay in the tracheostomy group was significantly longer than that in the non-tracheostomy group (25.5 (11–45) vs 20 (12–34) days, *P* = 0.0375), but the length of ICU stay was similar (12 (6–24) vs 12 (8–20) days, *P* = 0.2735). While 28-day mortality was significantly lower in patients that underwent tracheostomy (22.4% vs 30.3%), there was no significant difference between groups in 60-day (29.0% vs 34.6%, *P* = 0.06) or 90-day (30.3% vs 35.6%, *P* = 0.09) mortality.

**Fig. 4:**
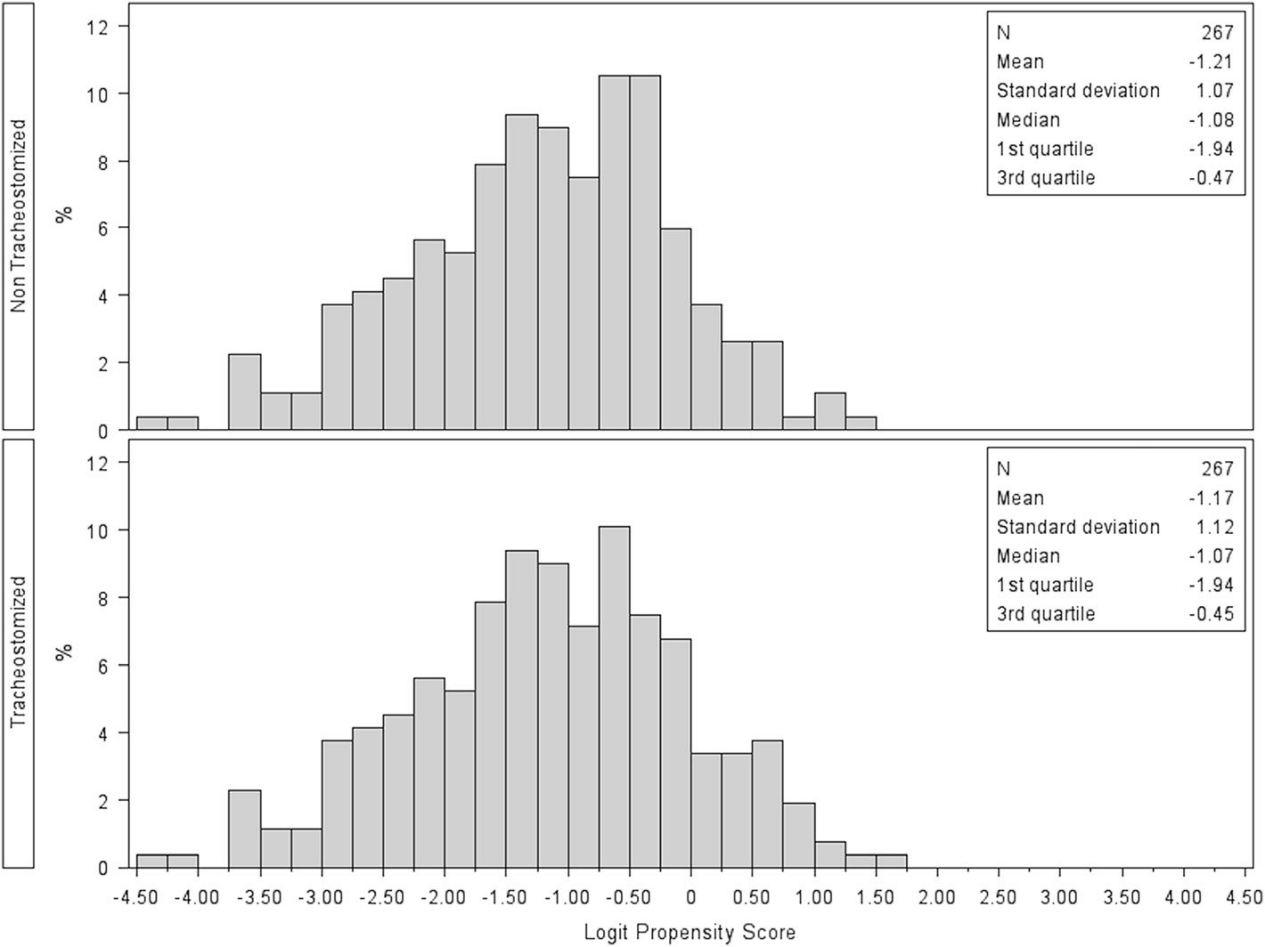
Distribution of the logit of propensity scores in patients with tracheostomy (*n* = 267) and without tracheostomy (*n* = 267) in the matched sample

**Fig. 5:**
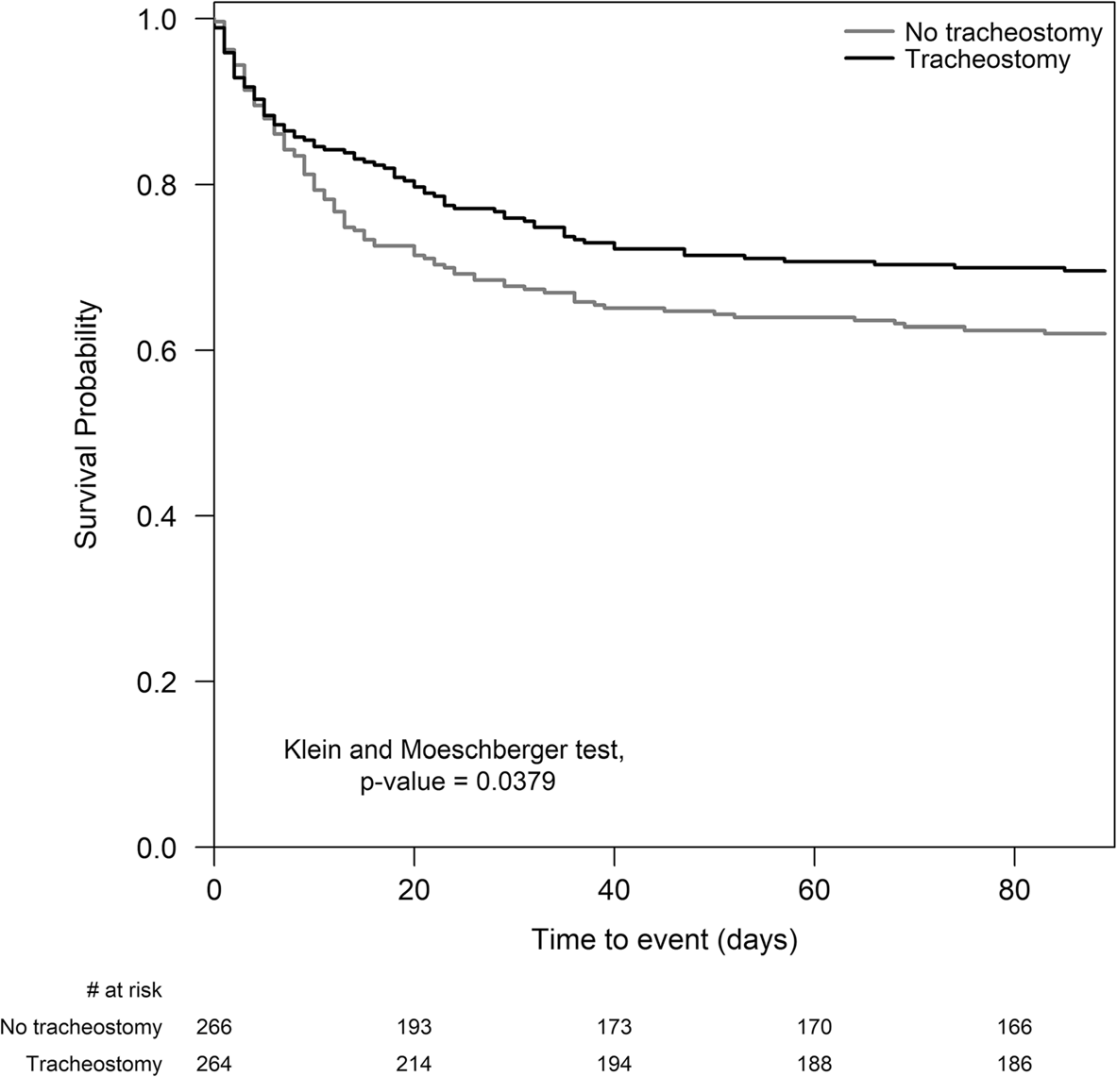
Survival probability during the hospital stay in the matched sample (*n* = 534). Kaplan Meier’s approach, assuming as censored, those patients discharged and alive before day 90

In the propensity-matched analysis, 534 patients with and without tracheostomy were matched (*n* = 267 per group). Table 3 lists the baseline characteristics of propensity-matched patients. There was no significant difference in the chosen covariates between matched tracheostomized patients and non-tracheostomized patients (all standardized differences < 0.1). The histograms of the logit of propensity scores showed the good quality of the matching procedure (Fig. 4). The duration of mechanical ventilation in the tracheostomy group was significantly longer than that in the non-tracheostomy group (median (Q1–Q3) 22 (12–33.5) days vs Q1–Q3 (4–12) days, *P* < 0.0001). The length of ICU and hospital stay in the tracheostomy group was significantly longer than that in the non-tracheostomy group (11 (5–24) vs Q1–Q3 (5-14) days, *P* < 0.0001 and 24 (9–43) vs 17 (10–31) days, *P* = 0.0190, respectively). Survival analysis showed that patients receiving tracheostomy had higher survival probability during the follow-up time compared to those without it (Klein and Moeschberger test, *P* = 0.0379) (Fig. 5). While the 28-day mortality was lower in the tracheostomy group as compared with non-tracheostomy group (22.9% vs 31.8%, *P* = 0.02) (Table 4), the 60-day (29.3% vs 36.3%, *P* = 0.08) or 90-day (30.5% vs 38.2%, *P* = 0.055) mortality ratio was not significantly different between tracheostomized and non-tracheostomized patients.

**Table 3:** Description of each covariate used for the propensity-score matching in the matched sample (*n* = 534)

	Tracheostomy	No tracheostomy	Standardized differences of mean
	Number (%) or mean ± sd	Number (%) or mean ± sd	
Number	267	267	
Age	58.4 ± 16.6	58.9 ± 17.9	0.03
Sex (male)	171 (64.0)	171 (64.0)	0.00
BMI	27.9 ± 7.8	27.9 ± 15.0	0.00
Geographic area			
European countries with high income	174 (64.2)	172 (64.4)	0.02
Non-European countries with high income	49 (18.4)	48 (18.0)	0.01
Countries with middle income	44 (16.5)	47 (17.6)	0.03
Type of admission			
Medical	178 (66.7)	183 (68.5)	0.04
Surgical	52 (19.5)	48 (18.0)	0.04
Elective	19 (7.1)	21 (7.9)	0.03
Trauma	18 (6.7)	15 (5.6)	0.05
Comorbidities			
COPD or home ventilation	60 (22.5)	65 (24.3)	0.04
Diabetes mellitus	64 (23.9)	64 (23.9)	0.00
Chronic renal failure	27 (10.1)	23 (8.6)	0.05
Immunosuppression or active or hematologic neoplasm	66 (24.7)	62 (23.3)	0.04
Heart failure (NYHA classes III-IV)	19 (7.1)	22 (8.2)	0.04
Chronic liver failure (Child-Pugh Class C)	2 (0.7)	2 (0.7)	0.00
Cause of AHRF - pneumonia	186 (69.7)	195 (73.0)	0.07
ARDS risk factor			
No risk factor	17 (6.4)	17 (6.4)	0.00
Only indirect risks factor	45 (16.9)	39 (14.6)	0.06
Only direct risk factors	168 (62.9)	173 (65.0)	0.04
Both risk factors	37 (13.9)	38 (14.2)	0.01
ECMO use	19 (7.1)	16 (6.0)	0.05
Arterial gas			
pH	7.4 ± 0.1	7.4 ± 0.1	0.07
P/F ratio (mmHg)	204.1 ± 86.5	199.1 ± 63.7	0.07
PaCO2 (mmHg)	44.4 ± 13.9	44.2 ± 14.1	0.01
Non-respiratory SOFA score adjusted for missing values,	5.1 ± 3.4	5.1 ± 3.6	0.00

**Table 4:** Description of outcomes in the propensity-matched sample (n = 534)

	Tracheostomy (*n* = 267)	No tracheostomy (*n* = 267)	*P* value
	Number (%) or median (Q1–Q3)	Number (%) or median (Q1–Q3)	
Days of mechanical ventilation			
All patients	22 [13–33.5]	8 [4–12]	<.0001
Patient alive at hospital discharge	22 [13.5–33]	6 [3–11]	<.0001
Ventilator-free days			
All patients	0 [0–11]	18 [0–25]	<.0001
Patient alive at hospital discharge	7 [0–15.5]	23 [18–26]	<.0001
Length of ICU stay (days)°			
All patients	11 [5–24]	8 [5–14]	<.0001
Patient alive at hospital discharge	12 [6–25]	8 [5–14]	0.0002
Length of hospital stay (days)°			
All patients	24 [9–43]	17 [10–31]	0.0190
Patient alive at hospital discharge	31 [15.5–50.5]	23 [13–38]	0.0325
Hospital mortality			
28-day*	61 (22.9)	85 (31.8)	0.0197
60-day*	78 (29.3)	97 (36.3)	0.0814
90-day*	81 (30.5)	102 (38.2)	0.0549
Limitation of life-sustaining therapies or measures decision	53 (19.9)	59 (22.1)	0.5900

Statistical tests accounted for the matched nature of the sample (paired *t* test or Wilcoxon signed-rank test for continuous variables, McNemar’s test for dichotomous variables)

°For tracheostomized patients, length of stay was valuated from the “approximate” date of tracheostomy

*Mortality was evaluated according to the vital status at 28/60/90 days from acute respiratory distress syndrome onset or from the “nearest recorded” date of tracheostomy for non-tracheostomized and tracheostomized patients, respectively. If the patient was discharged alive before 28/60/90 days, we considered the patient as alive

## Discussion

Tracheostomy was performed in 13% of patients with ARDS that were recruited to the LUNG SAFE international, multicenter, prospective cohort study from 459 ICUs across 50 countries, predominantly (75%) after the first week of their critical illness. Patients with tracheostomy remained longer on mechanical ventilation, and stayed longer in the ICU and in the hospital than non-tracheostomized patients. While duration of survival was increased in patients that received a tracheostomy, there was no significant increase in 60-day or 90-day survival compared to patients that did not receive a tracheostomy.

### Use of and timing of tracheostomy

The rate of tracheostomy in our cohort, 13%, is higher than that reported from a nationwide population-based study from the USA, in which 9.1% of all mechanically ventilated patients underwent tracheostomy [7]. The higher rate in our cohort could be explained by the inclusion of more patients with a severe form of respiratory failure, namely ARDS. Whether it should be performed earlier versus later is controversial [12]. While some studies reported that early tracheostomy may be related to better outcomes [3, 7], a recent large-scale randomized trial, TracMan, and a subsequent meta-analysis did not confirm these findings [11, 13]. The TracMan study demonstrated that early tracheostomy (within 4 or 5 days of critical care admission) did not improve mortality.

In our study, the median time to tracheostomy was 14 days, similar to that reported in other recent studies [7, 14]. Only a quarter of tracheostomies were performed on or before day 7. Of the 15% of patients who received tracheostomy on day 1, a substantial proportion may have had an indication for a surgical airway (e.g. for upper airway obstruction). Excluding these patients would even strengthen the trend toward later tracheostomy. These findings on tracheostomy timing represent a significant practice change, likely due to the findings of the TracMan study [11].

### Indications for tracheostomy

Medical indications for tracheostomy include the need for prolonged mechanical ventilation, need for airway access for secretion removal, and improvement of patient comfort [15]. A common reason for tracheostomy is the (clinician-predicted) likelihood that the patient will require prolonged mechanical ventilation. Of interest, the TracMan study demonstrated the difficulty for clinicians in predicting which patients will require prolonged ventilation support in the early phases of critical illness, given that many of their patients randomized to later tracheostomy did not receive one. In the present study, patients that received tracheostomy had fewer ventilator-free days, and required longer ICU and hospital stays. This is not to suggest that tracheotomy delayed weaning from ventilation, but is more likely a reflection of the fact that the patients that received tracheostomy were appropriately selected as being a group that would require prolonged ventilatory support.

There was considerable variation in the use of tracheostomy by geo-economic region, suggesting there are important regional and/or socio-economic differences in clinician use of tracheostomy in patients with ARDS. We used the 2016 World Bank countries socio-economic classification, which includes data on gross national income per person, to define three major geo-economic groupings as in a previous report [10]. In high-income Europe countries, more patients underwent tracheostomy than in the other regions (non-European countries (rest of world) with high income or countries with middle income). Our findings [7] indicate that the likelihood of a patient receiving a tracheostomy appears to be influenced by factors other than those that are related to their clinical status, such as local medical practices and expertise and costs relating to the procedure and equipment.

Of interest, our study did not replicate the findings of a US study, which showed a difference in indication by patient characteristics such as gender and race/ethnicity [7]. Because our study includes data from ICUs of numerous countries and not just the USA, it might represent a more generalizable picture of worldwide patterns in the use of tracheostomy.

### Patient outcomes

Aside from emergency situations, tracheostomy is usually performed in patients who are clinically relatively stable, but likely to need prolonged ventilation. This fact means that direct comparison of patients that received a tracheostomy to those that did not may be confounded by two important sources of bias, namely bias by indication (i.e. tracheostomy is more likely to be performed in “stable” patients) and immortal time bias (patients that die early are less likely to receive a tracheostomy). To address the latter issue, in our direct comparison of patients that received tracheostomy to those that did not, we calculated survival duration using the day of insertion of tracheostomy as “day 0”. Despite this “correction”, our data demonstrated significantly higher 28-day, 60-day and 90-day survival in patients that received tracheostomy.

Our subsequent analyses were designed to understand, and further minimize the potential for both types of bias. First, we stratified patients into subgroups to differentiate those who were in the ICU for at least 5 days, to reduce the impact of early death on outcomes. In this analysis, while 28-day mortality remained significantly lower in patients with tracheostomy, the trend to better 60-day and 90-day survival was not significant. These latter findings are confirmed in a propensity-matched analyses; patients undergoing tracheostomy had better 28-day, but not 60-day or 90-day survival. This is an important finding, as it suggests that while tracheostomy prolongs short-term survival, it may not improve longer-term outcomes.

Our finding is supported by a recent systematic review and meta-analysis that suggested there was no significant difference between early and late or no tracheostomy for length of hospital stay and long-term outcomes [8], although it was not a direct comparison between patients that received a tracheostomy and those that did not. Interestingly, another population-based US study reported conflicting results [7], in that their patients with tracheostomy had shorter lengths of stay. However, the authors raised the possibility that this finding could be due to patients being discharged to long-term care facilities because of pressures to reduce length of hospital stay. They considered that the place of death could have merely shifted from hospitals to long-term care facilities [7]. This finding underlines the need for studies that seek to determine the effect of tracheostomy on outcome to examine longer-terms outcomes, including follow up of patients post hospital discharge.

### Limitations

Several limitations of this study should be acknowledged. First, our study was an observational study and as such, we cannot make causal inferences. Second, the study period was relatively short and confined to the winter season, which might have led to sampling bias. However, while winter is the epidemic season of respiratory diseases such as influenza [16], the nature of the pathophysiology of ARDS does not have a significant seasonal variation. For example, seasonal variability of ARDS prevalence was modest in the recently published APRONET study [17]. A third limitation is that the group of patients who received early tracheostomy might have included patients who did not need tracheostomy. However, early tracheostomy was performed relatively infrequently in this study compared to previous studies [11]. Fourth, despite our efforts to address bias as discussed previously, the potential remains for bias by indication, and for immortal time bias to affect our results. Additional analyses, including confining the analyses to patients that were alive and in the ICU at 1 week, did not change the findings, confirming the robustness of the current findings. Although differences in goals of care may influence the tracheostomy decision, we did not find any difference in regard to end-of-life decision-making between the groups in our propensity-matched analysis, suggesting that this did not have an influence. Fifth, we did not have data on the methods of tracheostomy insertion, airway management methods, tracheostomy-related complications, and whether any deaths resulted from the tracheostomy itself is a limitation. Sixth, to control immoral bias, we chose the a cutoff point of early tracheostomy was day 4 from just TrachMan study [11]. As Fig. 2 demonstrates, moving the cutoff beyond day 5 has a limited effect: only two patients would be shifted if we changed the day of tracheostomy from day 5 to day 7. Only five patients would be shifted even if we changed the day of tracheostomy form day 5 to day 10. Finally, a longer follow-up period (180 days or more) might be beneficial in helping understand the long-term outcomes in patients on prolonged mechanical ventilation.

## Conclusions

In this international, multicenter, prospective cohort study, tracheostomy was performed in 13% of patients with ARDS, and was performed predominantly (in 75%) after the first week of their critical illness. Patients with tracheostomy remained longer on mechanical ventilation, and stayed longer in the ICU, and in the hospital than non-tracheostomized patients. While duration of survival was increased in patients that received a tracheostomy, there was no significant increase in 60-day or 90-day survival, suggesting that tracheostomy may delay death but does not impact longer term survival.

## Key messages

- Tracheostomy was performed in 13% of patients with ARDS, and was performed predominantly (in 75%) after the first week of their critical illness.
- Patients with tracheostomy remained longer on mechanical ventilation, and stayed longer in the ICU, and in the hospital than non-tracheostomized patients.
- While duration of survival was increased in patients that received a tracheostomy, there was no significant increase in 60-day or 90-day survival.
- Tracheostomy might delay - rather than prevent - death in some patients with ARDS.

## Additional files

Additional file 1: **Table S1.** Additional baseline characteristics in patients with tracheostomy and no tracheostomy (*n* = 2377). BMI, body mass index; ICU, intensive care unit; ER, emergency room; COPD, chronic obstructive pulmonary disease; NYHA, New York heart association; AHRF, Acute hypoxemic respiratory failure; ARDS, acute respiratory distress syndrome; TRALI, transfusion-related acute lung injury; A/C, assist control; PC, pressure control; BIPAP, bilevel positive airway pressure APRV, airway pressure release ventilation; SIMV, synchronized intermittent mandatory ventilation, PRVC, pressure-regulated volume control; PSV, pressure support ventilation; HFO, high-frequency oscillation; CPAP, continuous positive airway pressure IBW, ideal body weight; PEEP, positive end-expiratory pressure; ECMO, extracorporeal membrane oxygenation; SOFA, sequential organ failure assessment. Missing data: source of admission to ICU = 1, Chest x-ray/CT scan number = 1. (DOCX 27 kb)

Additional file 2: **Table S2** compares outcomes between early (within 7 days of ICU admission) and late (8 days and later) thoracotomy (*n* = 280). SD, standard deviation; ICU, intensive care unit; Q1–Q3; 25%–75% interquartile. Missing data: days of mechanical ventilation = 37; days of mechanical ventilation in patient alive at hospital discharge (90 days) = 139; length of ICU stay in patient alive at ICU discharge (90 days) = 58; length of hospital stay = 19; length of hospital stay in patient alive at ICU discharge (90 days) = 87; ICU, 28-day, 60-day, and 90-day mortality = 1. Participants were adult patients (≥ 18 years) with severe or moderate ARDS who received mechanical ventilation and had tracheostomy. Participants were excluded if they had made the decision to withhold/withdraw treatment; if they had been transferred from another hospital with invasive mechanical ventilation; if they received tracheostomy on the first day of the study period and had been on invasive mechanical ventilation for 6 days or more; or if they had been discharged from the ICU or died in the ICU within 7 days. Length of ICU and hospital stay were calculated from their admission to discharge. Mortality was calculated from day 7 to patient discharge. Days of mechanical ventilation, length of ICU stay, and length of hospital stay were compared using linear regression models, and mortality using logistic regression models. (DOCX 17 kb)

## Abbreviations

ARDS: Acute respiratory distress syndrome
BMI: Body mass index
ECMO: Extracorporeal membrane oxygenation
ESICM: European Society of Intensive Care Medicine
ICUs: Intensive care units
LUNG-SAFE: Large observational study to understand the global impact of severe acute respiratory failure
PaCO2: Partial arterial pressure of carbon dioxide
rWORLD: Non-European rest of world high income countries
SES: Socio-economic status
SOFA: Sequential organ failure assessment
VFDs: Ventilator-free days

## References

[1] Jaeger JM, Littlewood KA, Durbin CG (2002). The role of tracheostomy in weaning from mechanical ventilation. Respir Care.

[2] Mahmood K, Wahidi MM (2016). The changing role for tracheostomy in patients requiring mechanical ventilation. Clin Chest Med.

[3] Hosokawa K, Nishimura M, Egi M, Vincent JL (2015). Timing of tracheotomy in ICU patients: a systematic review of randomized controlled trials. Crit Care.

[4] Laffey JG, Bellani G, Pham T, Fan E, Madotto F, Bajwa EK, Brochard L, Clarkson K, Esteban A, Gattinoni L (2016). Potentially modifiable factors contributing to outcome from acute respiratory distress syndrome: the LUNG SAFE study. Intensive Care Med.

[5] Bellani G, Laffey JG, Pham T, Fan E, Brochard L, Esteban A, Gattinoni L, van Haren F, Larsson A, McAuley DF (2016). Epidemiology, patterns of care, and mortality for patients with acute respiratory distress syndrome in intensive care units in 50 countries. JAMA.

[6] Groves DS, Durbin CG (2007). Tracheostomy in the critically ill: indications, timing and techniques. Curr Opin Crit Care.

[7] Mehta AB, Syeda SN, Bajpayee L, Cooke CR, Walkey AJ, Wiener RS (2015). Trends in tracheostomy for mechanically ventilated patients in the United States, 1993-2012. Am J Respir Crit Care Med.

[8] Siempos II, Ntaidou TK, Filippidis FT, Choi AM (2015). Effect of early versus late or no tracheostomy on mortality and pneumonia of critically ill patients receiving mechanical ventilation: a systematic review and meta-analysis. Lancet Respir Med.

[9] Andriolo BN, Andriolo RB, Saconato H, Atallah AN, Valente O (2015). Early versus late tracheostomy for critically ill patients. Cochrane Database Syst Rev.

[10] Laffey JG, Madotto F, Bellani G, Pham T, Fan E, Brochard L, Amin P, Arabi Y, Bajwa EK, Bruhn A (2017). Geo-economic variations in epidemiology, patterns of care, and outcomes in patients with acute respiratory distress syndrome: insights from the LUNG SAFE prospective cohort study. Lancet Respir Med.

[11] Young D, Harrison DA, Cuthbertson BH, Rowan K, TracMan C (2013). Effect of early vs late tracheostomy placement on survival in patients receiving mechanical ventilation: the TracMan randomized trial. JAMA.

[12] Cheung NH, Napolitano LM (2014). Tracheostomy: epidemiology, indications, timing, technique, and outcomes. Respir Care.

[13] Meng L, Wang C, Li J, Zhang J (2016). Early vs late tracheostomy in critically ill patients: a systematic review and meta-analysis. Clin Respir J.

[14] Esteban A, Anzueto A, Alia I, Gordo F, Apezteguia C, Palizas F, Cide D, Goldwaser R, Soto L, Bugedo G (2000). How is mechanical ventilation employed in the intensive care unit? An international utilization review. Am J Respir Crit Care Med.

[15] Durbin CG (2005). Indications for and timing of tracheostomy. Respir Care.

[16] Ortiz JR, Neuzil KM, Shay DK, Rue TC, Neradilek MB, Zhou H, Seymour CW, Hooper LG, Cheng PY, Goss CH (2014). The burden of influenza-associated critical illness hospitalizations. Crit Care Med.

[17] Guerin C, Beuret P, Constantin JM, Bellani G, Garcia-Olivares P, Roca O, Meertens JH, Maia PA, Becher T, Peterson J (2018). A prospective international observational prevalence study on prone positioning of ARDS patients: the APRONET (ARDS prone position network) study. Intensive Care Med.

