# Supplementary material for: Epidemiology and patterns of tracheostomy practice in patients with acute respiratory distress syndrome in ICUs across 50 countries
Source: Crit Care. 2018 Aug 17;22:195. doi: 10.1186/s13054-018-2126-6 (PMC6097245; doi:10.1186/s13054-018-2126-6)
Supplement: Supplementary file 1 — Table S1. Additional baseline characteristics in patients with tracheostomy and no tracheostomy (n = 2377). BMI, body mass index; ICU, intensive care unit; ER, emergency room; COPD, chronic obstructive pulmonary disease; NYHA, New York heart association; AHRF, Acute hypoxemic respiratory failure; ARDS, acute respiratory distress syndrome; TRALI, transfusion-related acute lung injury; A/C, assist control; PC, pressure control; BIPAP, bilevel positive airway pressure APRV, airway pressure release ventilation; SIMV, synchronized intermittent mandatory ventilation, PRVC, pressure-regulated volume control; PSV, pressure support ventilation; HFO, high-frequency oscillation; CPAP, continuous positive airway pressure IBW, ideal body weight; PEEP, positive end-expiratory pressure; ECMO, extracorporeal membrane oxygenation; SOFA, sequential organ failure assessment. Missing data: source of admission to ICU = 1, Chest x-ray/CT scan number = 1. (DOCX 27 kb) [file 13054_2018_2126_MOESM1_ESM.docx]

Table S1 Additional baseline characteristics between patients with tracheostomy and no tracheostomy. (n=2,377)

|  | **Tracheostomy** (n=309)  n (%) or  median (Q1-Q3) | **No tracheostomy**  (n=2,068)  n (%) or  median (Q1-Q3) | P value |
| --- | --- | --- | --- |
| ***Source of admission to ICU***  Other hospital (ICU)  Other hospital (Ward)  Study Hospital (Ward)  Study Hospital (Other ICU)  Operation room  ER/ambulance  Other | 53 (17.5)  21 (6.8)  73 (23.6)  6 (1.9)  32 (10.4)  112 (36.3)  12 (3.9) | 175 (8.5)  185 (9.0)  575 (27.8)  58 (2.8)  216 (10.5)  798 (38.6)  60 (2.9) | 0.0002 |
| ***Comorbidities***  COPD  Home ventilation  Diabetes mellitus  Chronic renal failure  Active Neoplasm  Hematologic neoplasm  Immunosuppression  Heart failure (NYHA classes III-IV)  Chronic liver failure (Child-Pugh Class C) | 65 (21.0)  6 (1.9)  72 (23.3)  30 (9.7)  24 (7.8)  12 (3.9)  48 (15.5)  20 (6.5)  4 (1.3) | 407 (19.7)  32 (1.6)  443 (21.4)  194 (9.4)  175 (8.5)  95 (4.6)  242 (11.7)  204 (9.9)  99 (4.8) | 0.5777  0.6242  0.4545  0.8541  0.6806  0.5743  0.0549  0.0570  0.0049 |
| ***ARDS risk factor***  DIRECT  Pneumonia  Aspiration of gastric contents  Inhalational injury  Pulmonary injury  Pulmonary vasculitis  Drowning  INDIRECT  Non-pulmonary sepsis  Major trauma  Pancreatitis  Severe burns  Noncardiac shock  Drug overdose  TRALI  Others | 202 (65.4)  38 (12.3)  4 (1.3)  16 (5.2)  1 (0.3)  0 (0)  42 (13.6)  21 (6.8)  1 (0.3)  1 (0.3)  23 (7.4)  2 (0.7)  16 (5.2)  11 (3.6) | 1,183 (57.2)  334 (16.1)  64 (3.1)  64 (3.1)  8 (0.4)  2 (0.1)  371 (17.9)  86 (4.2)  49 (2.4)  7 (0.3)  176 (8.5)  47 (2.3)  87 (4.2)  58 (2.8) | 0.0066  0.0821  0.0766  0.0582  1.0000  1.0000  0.0599  0.0370  0.0194  1.0000  0.5275  0.0607  0.4342  0.4608 |
| ***Chest X-ray/CT scan number of involved quadrants at day 1***  0  1  2  3  4 | 0 (0)  0 (0)  113 (36.6)  76 (24.6)  120 (38.8) | 2 (0.1)  31 (1.5)  865 (41.9)  459 (22.2)  710 (34.4) | 0.0382 |
| ***Modality of mechanical ventilation at ARDS onset***  Ventilator setting at ARDS onset  Mode  A/C  PC/BIPAP/APRV  SIMV  PRVC  PSV  HFO  CPAP  Others | 80 (25.9)  98 (31.7)  39 (12.6)  28 (9.1)  49 (15.9)  0 (0)  10 (3.2)  5 (1.6) | 757 (36.6)  489 (23.7)  353 (17.1)  185 (9.0)  201 (9.7)  2 (0.1)  45 (2.2)  36 (1.7) | <.0001 |

BMI, body mass index; ICU, intensive care unit; ER, emergency room; COPD, chronic obstructive pulmonary disease; NYHA, New York heart association; AHRF, Acute hypoxemic respiratory failure; ARDS, acute respiratory distress syndrome; TRALI, transfusion-related acute lung injury; A/C, assist control; PC, pressure control; BIPAP, bilevel positive airway pressure APRV, airway pressure release ventilation; SIMV, synchronized intermittent mandatory ventilation, PRVC, pressure regulated volume control; PSV, pressure support ventilation; HFO, high-frequency oscillation; CPAP, continuous positive airway pressure IBW, ideal body weight; PEEP, positive end expiratory pressure; ECMO, extracorporeal membrane oxygenation; SOFA, sequential organ failure assessment

Missing data: Source of admission to ICU=1, Chest X-ray/CT scan number=1.
