# Supplementary material for: Epidemiology and patterns of tracheostomy practice in patients with acute respiratory distress syndrome in ICUs across 50 countries
Source: Crit Care. 2018 Aug 17;22:195. doi: 10.1186/s13054-018-2126-6 (PMC6097245; doi:10.1186/s13054-018-2126-6)
Supplement: Supplementary file 2 — Table S2 compares outcomes between early (within 7 days of ICU admission) and late (8 days and later) thoracotomy (n = 280). SD, standard deviation; ICU, intensive care unit; Q1–Q3; 25%–75% interquartile. Missing data: days of mechanical ventilation = 37; days of mechanical ventilation in patient alive at hospital discharge (90 days) = 139; length of ICU stay in patient alive at ICU discharge (90 days) = 58; length of hospital stay = 19; length of hospital stay in patient alive at ICU discharge (90 days) = 87; ICU, 28-day, 60-day, and 90-day mortality = 1. Participants were adult patients (≥ 18 years) with severe or moderate ARDS who received mechanical ventilation and had tracheostomy. Participants were excluded if they had made the decision to withhold/withdraw treatment; if they had been transferred from another hospital with invasive mechanical ventilation; if they received tracheostomy on the first day of the study period and had been on invasive mechanical ventilation for 6 days or more; or if they had been discharged from the ICU or died in the ICU within 7 days. Length of ICU and hospital stay were calculated from their admission to discharge. Mortality was calculated from day 7 to patient discharge. Days of mechanical ventilation, length of ICU stay, and length of hospital stay were compared using linear regression models, and mortality using logistic regression models. (DOCX 17 kb) [file 13054_2018_2126_MOESM2_ESM.docx]

Table S2 compares outcomes between early (within 7 days of ICU admission) and late (8 days and later) thoracotomy (n=280)

|  | **Early tracheostomy**  (n=59)  n (%) or  median (Q1-Q3) | **Late tracheostomy**  (n=221)  n (%) or  median (Q1-Q3) | P value |
| --- | --- | --- | --- |
| Days of mechanical ventilation (n=51, n=192) | 14 (10-26) | 24 (16.5-37.0) | <.0001 |
| Days of mechanical ventilation in patient alive at hospital discharge (90 days) (n=35, n=139) | 15 (11-27) | 24 (16-37) | 0.0007 |
| Length of ICU stay | 16 (12-26) | 29 (20-43) | <.0001 |
| Length of ICU stay in patient alive at ICU discharge (90 days) (n=47, n=175) | 17 (13-29) | 29 (20-43) | <.0001 |
| Length of hospital stay (n=57, n=204) | 28 (17-46) | 41.5 (26-64) | 0.0001 |
| Length of hospital stay in patient alive at ICU discharge (90 days) (n=41, n=152) | 31 (24-46) | 50 (31-72.5) | 0.0006 |
| ICU mortality | 12/59 (20.3) | 45/220 (20.5) | 0.9844 |
| 28-day mortality | 13/59 (22.0) | 24/220 (10.9) | 0.0253 |
| 60-day mortality | 15/59 (25.4) | 49/220 (22.3) | 0.6092 |
| 90-day mortality | 16/59 (27.1) | 52/220 (23.6) | 0.5801 |

SD, standard deviation; ICU, intensive care unit; Q1-Q3; 25%-75% interquartile

Missing data: days of mechanical ventilation=37; days of mechanical ventilation in patient alive at hospital discharge (90 days)=139; length of ICU stay in patient alive at ICU discharge (90 days)=58; length of hospital stay=19; length of hospital stay in patient alive at ICU discharge (90 days)=87; ICU, 28-day, 60-day, and 90-day mortalities=1

Participants were adult patients (≥18 years) with severe or moderate ARDS who received mechanical ventilation and had tracheotomy. Participants were excluded if they had made the decision to withhold/withdraw treatment; if they had been transferred from another hospital with invasive mechanical ventilation; if they received tracheostomy on the first day of study-period and had been on invasive mechanical ventilation for 6 days or more; or if they had been discharged from the ICU or died in the ICU within 7 days. Length of ICU and hospital stay were calculated from their admission to discharge. Mortalities were calculated from day 7 to their discharge. Days of mechanical ventilation, length of ICU stay, and length of hospital stay were compared with liner regression models; mortalities with logistic regression models.
